# Supplementary material for: Structural Adaptive, Self-Separating Material for Removing Ibuprofen from Waters and Sewage
Source: Materials (Basel). 2021 Dec 13;14(24):7697. doi: 10.3390/ma14247697 (PMC8709425; doi:10.3390/ma14247697)
Supplement: Supplementary file 1 [file materials-14-07697-s001.zip › materials-1473594-supplementary.pdf]

Supplementary Materials

Structural Adaptive, Self−Separating Material for Removing Ibuprofen from Waters and Sewage

Anna Maria Skwierawska [1,*], Dominika Nowacka [1], Paulina Nowicka [1] and Sandra Rosa [1] and Katarzyna Kozłowska-Tylingo [2]

[1] Department of Chemistry and Technology of Functional Materials, Gdansk University of Technology, Narutowicza 11/12, 80−233 Gdansk, Poland; dnowacka97@gmail.com (D.N.); paulina.nowicka1234@gmail.com (P.N.); sandrarosaaaa@gmail.com (S.R.)
[2] Department of Pharmaceutical Technology and Biochemistry, Gdansk University of Technology, Narutowicza 11/12, 80−233 Gdansk, Poland; katarzyna.kozlowska-tylingo@pg.edu.pl
* Correspondence: anna.skwierawska@pg.edu.pl

Table S1. The reported concentrations of IBU (ng L^{-1}) in waters.

Country	River	Concentration	References
France	NA	4.5	[1]
Greece	Aisonas	17	[2]
Mexico	−	1406	[3]
Portugal	Lima River	723	[4]
USA	Mississippi	34	[5]

Country	Concentration in Raw Sewage	Concentration in Treated Sewage	Removal Efficiency [%]	References
Poland (Hajdów Lublin)	649	412	37	[6]
Poland (Gdańsk)	1664	679	59	[6]
Poland (Szczecin)	894	516	42	[6]
USA (Maryland)	9500–14700	10–20	99	[7]
Switzerland	2000–3000	600–800	53–79	[7]
United Kingdom	5700	180	97	[8]
Scotland	28000	3000	98	[9]
Algeria	1607.8	341.4	79	[10]

Country	Concentration	References
France	6	[1]
Algeria	142.1	[10]
Kolumbia	7–58	[11]

S1. Material characterization (β−CD−M)

Elemental Analysis

Preparation of samples for elemental analysis consisted in drying at the temperature of 100 °C in a moisture analyzer until a constant mass was obtained. The powders were immediately transferred into packages, which were sealed and sent to the elemental analysis laboratory.

The basic element of the material most likely contains three β−CD molecules. One has as many as six substituted hydroxyl groups, while the other two only have five. Fourteen hydroxyl groups were involved in the usual polyurethane bonds while two others form the allophanate system, which explains the high nitrogen content.

Anal. Calcd. (β−CD−M): $C_{262}H_{434}N_{36}O_{141}$: 7.95 (N), 49.60 (C), 6.89 (H), 35.56 (O).

Anal. found. (β−CD−M): 7.93 (N), 49.26 (C), 6.84 (H), 35.05 (O).

Table S2. Selected FTIR bands recorded during the analysis of raw material and product samples (KBr pellet).

Compound	Wavenumber (cm⁻¹)	Vibration Allocation
**β−CD**	3385.45 (strong, broad)	OH stretching intermolecular bonded
	2929.23 (medium, broad)	OH stretching intermolecular bonded
	1636.38 (medium, broad)	C=O stretching hemiacetal
	1412.06 (medium, broad)	OH−bending
	1157.02 – 1029.20 (strong, broad)	OH stretching
	1157.02 (sharp overtone)	OH stretching secondary alcohol
	1078.86 (Sharp overtone)	OH stretching primary alcohol
**HDI**	2939.74 (medium, broad)	C−H stretching methylene group
	2861.62 (medium, sharp)	C−H stretching methylene group
	2274.84 (strong, broad)	N=C=O stretching isocyanate
	1463.47 (sharp)	CH bending methylene group
	1355.46(sharp)	CH bending methylene group
	863.50 (sharp)	CH_2 rocking methylene group
	792.88(sharp)	CH_2 rocking methylene group
**β−CD−M**	3420.01(strong, broad)	OH stretching intermolecular bonded
	2934.19 (medium, broad)	C−H stretching alkane
	2860.08 (medium, sharp)	C−H stretching alkane
	1715.86 (strong, split)	C=O stretching urethane and allophanate
	1653.07, 1646.76 and 1636.22 (strong, split)	C=O stretching urethane and allophanate<br>C=O stretching hemiacetal
	1557.86, 1540.70, 1533.92 (strong, split)	C−N−H stretching urethane and allophanate
	1457.28 (split)	CH bending methylene group
	1418.01(split)	CH bending methylene group
	1260.48 (sharp)	OH stretching ester group C−(O)−O−C
	1151.52 – 1033.29 (strong, broad)	OH stretching
	1151.52 (sharp)	OH stretching secondary alcohol
	773.93(sharp)	CH_2 rocking methylene group

Table S3. List of selected FTIR bands recorded during the analysis of regenerated β−CD−M samples (KBr pellet).

Compound	Wavenumber (cm⁻¹)	Vibration allocation
**β−CD−M first regeneration**	34219.59 (strong, broad)	OH stretching intermolecular bonded
	2935.06 (medium, broad)	C−H stretching alkane
	2860.51 (medium, sharp)	C−H stretching alkane
	1705.72 (strong, split)	C=O stretching urethane allophanate
	16.53.07 and 1646.76<br>1636.22<br>(strong, split)	C=O stretching urethane and allophanate<br>C=O stretching hemiacetal
	1540.74 (strong, split)	C−N−H stretching urethane and allophanate
	1457.53 (split)	CH bending methylene group
	1417.47 (split)	CH bending methylene group
	1261.27 (sharp)	OH stretching ester group C−(O)−O−C
	1151.68–1031.97 (strong, broad)	OH stretching
	1151.68 (sharp)	OH stretching secondary alcohol
	774.33(sharp)	CH_2 rocking methylene group
**β−CD−M fith regeneration**	3415.26 (strong, broad)	OH stretching intermolecular bonded
	2933.77 (medium, broad)	C−H stretching alkane
	2859. 74 (medium, sharp)	C−H stretching alkane
	1709.26 (strong, split)	C=O stretching urethane and allophanate
	1651.57 (strong, split)	C=O stretching urethane and allophanate
	1546.18 (strong, split)	C−N−H stretching urethane and allophanate
	1461.12 (split)	CH bending methylene group
	1412.01(split)	CH bending methylene group
	1261.20 (sharp)	OH stretching ester group C−(O)−O−C
	1150.72–1033.37 (strong, broad)	OH stretching
	1150.72 (sharp)	OH stretching secondary alcohol
	773.62 (sharp)	CH_2 rocking methylene group

Preliminary Analysis of the Availability of Embedded β−CD Molecules in the Material

Buffer (0.05 M trisaminomethane hydrochloride—Tris HCl; pH = 7) was used to prepare standard β−CD solutions (0.1–0.5 mmol L^{-1}). Phenolphthaine working solution (0.6 mmol L^{-1}) was prepared by dissolving 1.5 ml of phenolphthaine stock solution (4 mmol L^{-1} in ethanol) in carbonate buffer at pH 10.5 just before the measurements. Test samples were prepared by mixing 1 mL of β−CD solution with 4 mL of phenolphthaine working solution. Separately, the 10 mg (20 mg) of material was suspended in a mixture of 1 ml of Tris HCl buffer and 4 ml of working phenolphthalein solution. All samples were shaken for one hour prior to absorbance measurements. The adsorbent was filtered off through Whatman Grade 540 filter paper. The absorbance of the standard solution and the samples was measured at 550 nm with a UV−VIS spectrophotometer (Hach Lange UV−vis DR 6000). A mixture of 1 ml of Tris HCl buffer and 4 ml of working phenolphthalein solution was used as a control solution. A calibration curve in form of absorbance as a function of β−CD weight was plotted for the standard solutions and an available for complexation β−CD was determined. Measurements were made in triplicate [12–14].

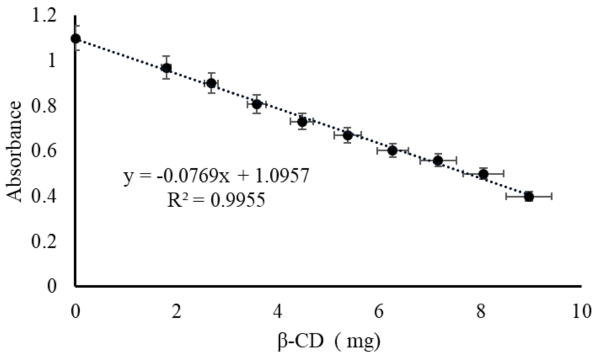

Figure S1. Spontaneous decrease in absorbance of phenolphthalein solution (λ = 550 nm).

Table S4. Changes in the value of phenolphthalein absorbance (A)in contact with 10 mg of β−CD−M.

β−CD−M (mg)	A (λ = 550 nm)	A Average
10	0.673	
10	0.668	0.672
10	0.675	

The amount of CD in the samples determined on the basis of the standard curve is 5.51 mg.

Specific Surface Area and Pore Size Distribution of Examined Adsorbent

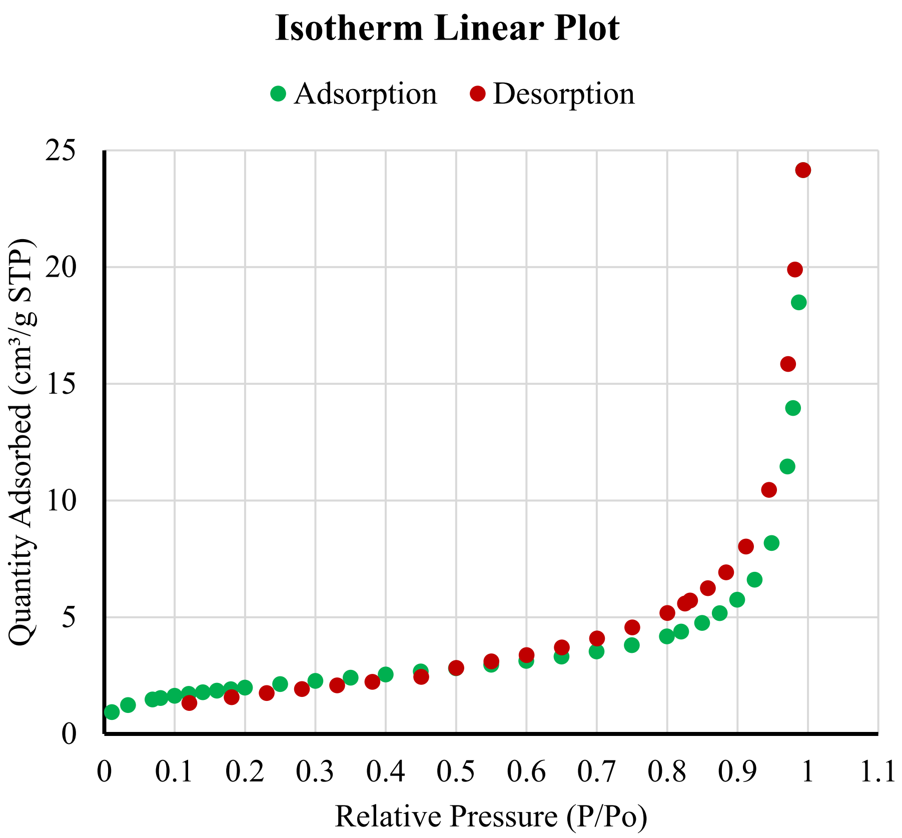

Figure S2. N_2 adsorption–desorption isotherms for β−CD−M at 77 K.

Table S5. BET Surface Area Report for β−CD−M.

Parameter	Value
BET Surface Area	7.3092 ± 0.0251 m²/g
Slope	0.585891 ± 0.002021 g/cm³ STP
Y−Intercept	0.009684 ± 0.000313 g/cm³ STP
C	61.499882
Qm	1.6790 cm³/g STP
Correlation Coefficient	0.9999583
Molecular Cross−Sectional Area	0.1620 nm²

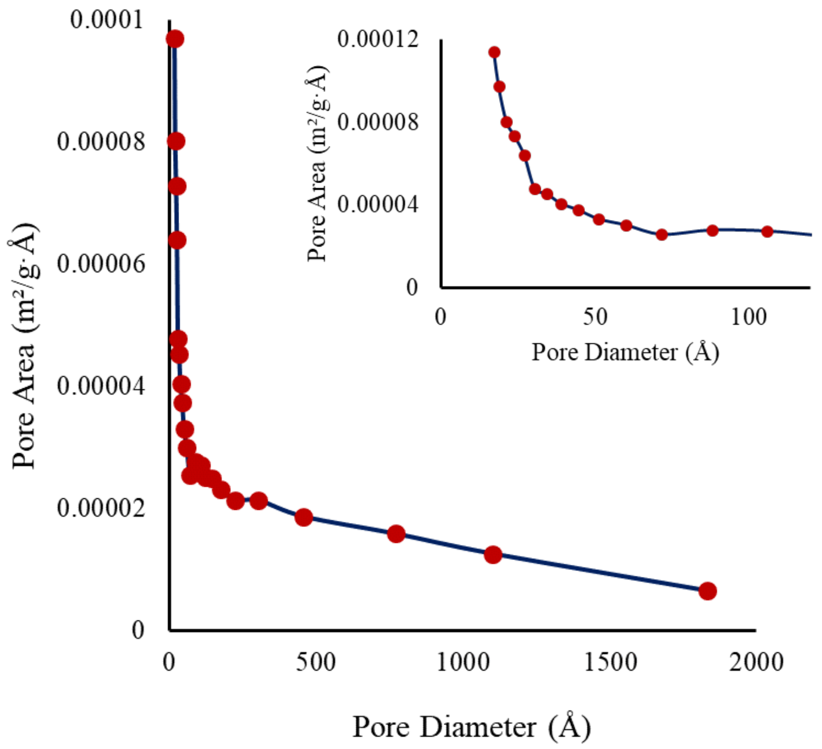

Figure S3. Barrett–Joyner–Halenda (BJH) pore size distribution from N_2 adsorption isotherm expressed as pore volume for β–CD–M.

Table S6. Porous property of β–CD–M.

Pore Diameter Range (Å)	Average Diameter (Å)	Incremental Pore Volume (cm³/g)	Cumulative Pore Volume (cm³/g)	Incremental Pore Area (m²/g)	Cumulative Pore Area (m²/g)
2947.2–1534.1	1830.774	0.009238	0.009238	0.201839	0.201839
1534.1–942.5	1102.189	0.007448	0.016686	0.270286	0.472125
942.5–682.2	770.697	0.004129	0.020815	0.214321	0.686446
682.2–387.8	457.6209	0.0055	0.026315	0.480775	1.167221
387.8–266.4	304.4597	0.002589	0.028905	0.340187	1.507408
266.4–201.4	224.6045	0.001389	0.030294	0.247348	1.754757
201.4–161.8	177.0667	0.000919	0.031213	0.20767	1.962427
161.8–135.0	145.8403	0.000666	0.031879	0.182658	2.145085
135.0–112.5	121.5831	0.000565	0.032444	0.185824	2.330908
112.5–100.9	106.0269	0.000315	0.032759	0.118879	2.449788
100.9–80.2	88.0325	0.000572	0.033331	0.259916	2.709704
80.2–66.2	71.72943	0.000359	0.03369	0.200382	2.910086
66.2–56.0	60.12403	0.000307	0.033997	0.204264	3.11435
56.0–48.1	51.39819	0.000258	0.034255	0.200487	3.314837
48.1–41.9	44.56554	0.000232	0.034487	0.208325	3.523163
41.9–36.9	39.0399	0.000206	0.034692	0.210607	3.733769
36.9–32.6	34.45013	0.000193	0.034885	0.22353	3.9573
32.6–29.0	30.5469	0.000175	0.03506	0.228849	4.186148
29.0–25.8	27.16056	0.000204	0.035264	0.300578	4.486727
25.8–22.9	24.15764	0.000208	0.035472	0.345152	4.831878
22.9–20.3	21.43404	0.00021	0.035682	0.391274	5.223152
20.3–17.9	18.91799	0.000236	0.035918	0.498134	5.721286
17.9–16.9	17.37733	0.000107	0.036025	0.246704	5.96799

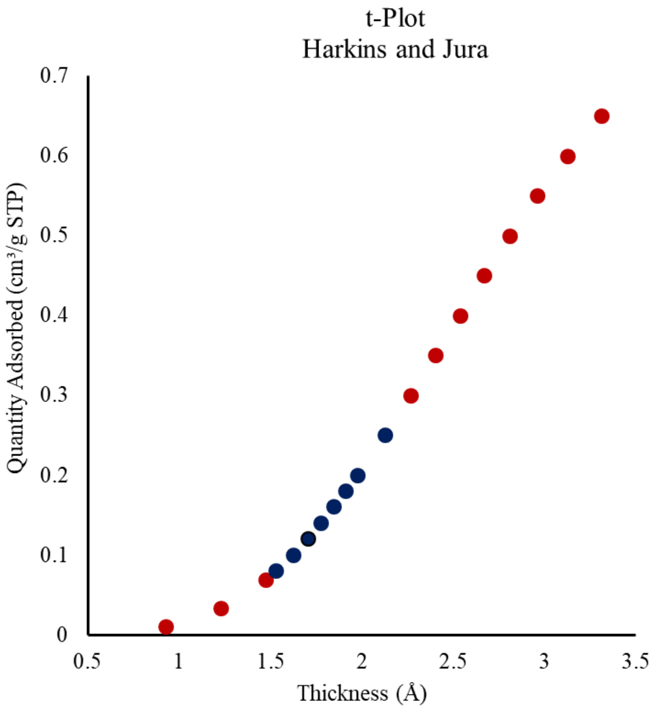

Figure S4. Harkins and Jura t−Plot for β−CD−M. Blue dots correspond to estimated isotherm, red represent not fitted points.

Table S7. t−Plot Report for β−CD−M.

**Parameter**	**Value**
Micropore Volume:	−0.000364 cm³/g
Micropore Area:	*
External Surface Area:	7.8226 m²/g
Slope:	0.505726 ± 0.008214 cm³/g·Å STP
Y−Intercept:	−0.235420 ± 0.033394 cm³/g STP
Correlation Coefficient:	0.999210
Surface Area Correction Factor:	1.000
Density Conversion Factor:	0.0015468
Total Surface Area (BET):	7.3092 m²/g
Thickness Range:	3.5000 Å to 5.0000 Å
Thickness Equation:	Harkins and Jura

*The micropore area is not reported because the micropore volume is negative.

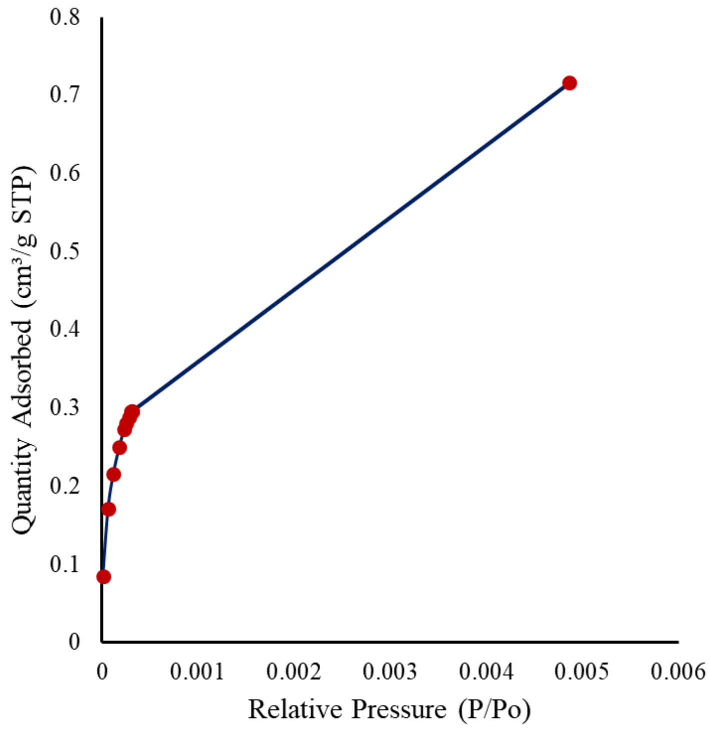

Figure S5. Horvath−Kawazoe Isotherm Linear Plot of β−CD−M.

Table S8. Summary rapport of β−CD−M microporous.

Parameter	Value
Volume in Pores<8.01 Å	0.00018 cm^3/g
Total Volume in Pores ≤ 19.43 Å	0.00118 cm^3/g
Area in Pores>19.43 Å	0.000 m^2/g
Total Area in Pores ≥ 8.01 Å	1.490 m^2/g
Maximum pore volume at P/P_0 = 0.004867606	0.001107 cm^3/g
Median pore width	8.415 Å

Thermogravimetric Analysis of β−CD−M

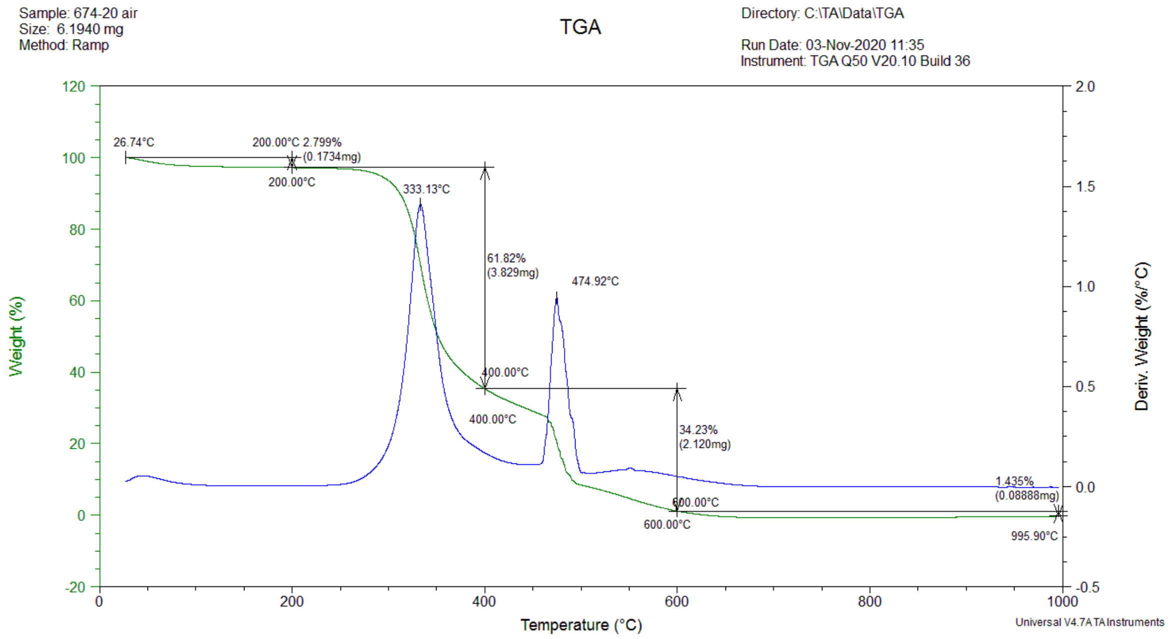

Figure S6. Thermogravimetric analysis of β−CD−M in the air atmosphere.

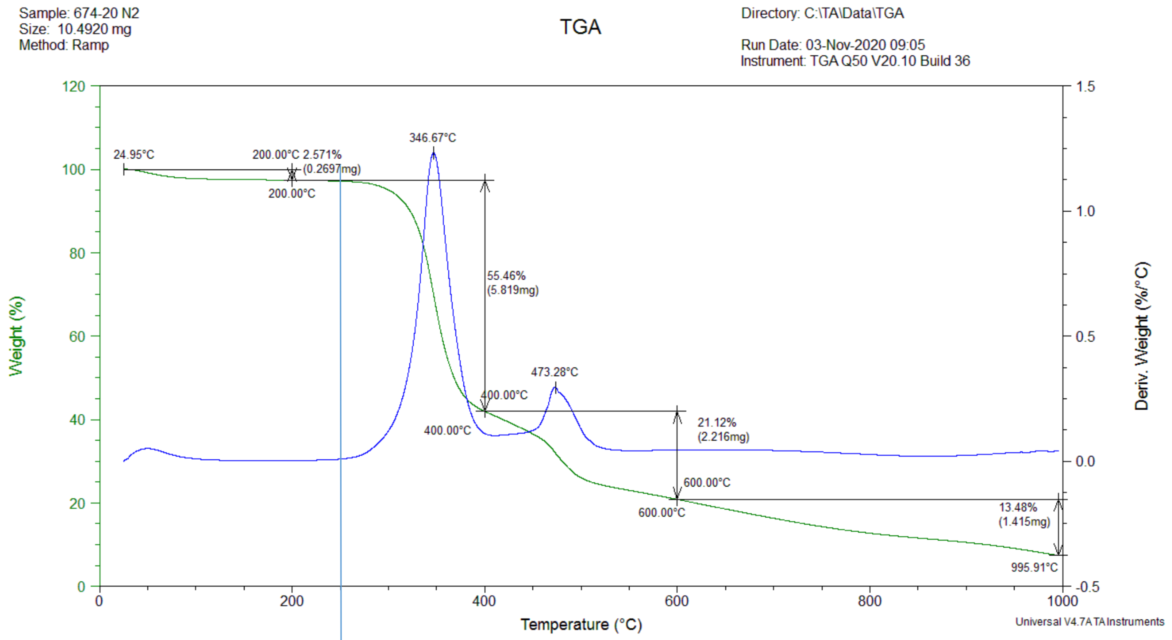

Figure S7. Thermogravimetric analysis of β−CD−M in nitrogen atmosphere.

References

1. Togola, A.; Budzinski, H. Multi-residue analysis of pharmaceutical compounds in aqueous samples. *J. Chromatogr. A* **2008**, *1177*, 150–158, doi:10.1016/j.chroma.2007.10.105.
2. Stasinakis, A.S.; Mermigka, S.; Samaras, V.G.; Farmaki, E.; Thomaidis, N. Occurrence of endocrine disrupters and selected pharmaceuticals in Aisonas River (Greece) and environmental risk assessment using hazard indexes. *Environ. Sci. Pollut. Res.* **2012**, *19*, 1574–1583, doi:10.1007/s11356-011-0661-7.
3. Gibson, R.; Durán-Álvarez, J.C.; Estrada, K.L.; Chávez, A.; Cisneros, B.J. Accumulation and leaching potential of some pharmaceuticals and potential endocrine disruptors in soils irrigated with wastewater in the Tula Valley, Mexico. *Chemosphere* **2010**, *81*, 1437–1445, doi:10.1016/j.chemosphere.2010.09.006.

4. Paíga, P.; Santos, L.; Amorim, C.G.; Araujo, A.; Montenegro, M.; Pena, A.; Delerue-Matos, C. Pilot monitoring study of ibuprofen in surface waters of north of Portugal. *Environ. Sci. Pollut. Res.* **2012**, *20*, 2410–2420, doi:10.1007/s11356-012-1128-1.
5. Zhang, S.; Zhang, Q.; Darisaw, S.; Ehie, O.; Wang, G. Simultaneous quantification of polycyclic aromatic hydrocarbons (PAHs), polychlorinated biphenyls (PCBs), and pharmaceuticals and personal care products (PPCPs) in Mississippi river water, in New Orleans, Louisiana, USA. *Chemosphere* **2007**, *66*, 1057–1069, doi:10.1016/j.chemosphere.2006.06.067.
6. Czerwiński, J.; Kłonica, A.; Ozonek, J.; Środowisku, W. Wodnym I Metody, Czaspomismo Inżynierii Lądowej, *Środowiska i Architektury*. **2015**, *62*, 27–42.
7. Tauxe-Wuersch, A.; De Alencastro, L.; Grandjean, D.; Tarradellas, J. Occurrence of several acidic drugs in sewage treatment plants in Switzerland and risk assessment. *Water Res.* **2005**, *39*, 1761–1772, doi:10.1016/j.watres.2005.03.003.
8. Quintana, J.B.; Weiss, S.; Reemtsma, T. Pathways and metabolites of microbial degradation of selected acidic pharmaceutical and their occurrence in municipal wastewater treated by a membrane bioreactor. *Water Res.* **2005**, *39*, 2654–2664, doi:10.1016/j.watres.2005.04.068.
9. Roberts, P.H.; Thomas, K.V. The occurrence of selected pharmaceuticals in wastewater effluent and surface waters of the lower Tyne catchment. *Sci. Total. Environ.* **2006**, *356*, 143–153, doi:10.1016/j.scitotenv.2005.04.031.
10. Kermia, A.E.B.; Fouial-Djebbar, D.; Trari, M. Occurrence, fate and removal efficiencies of pharmaceuticals in wastewater treatment plants (WWTPs) discharging in the coastal environment of Algiers. *Comptes Rendus Chim.* **2016**, *19*, 963–970, doi:10.1016/j.crci.2016.05.005.
11. Aristizabal-Ciro, C.; Botero-Coy, A.M.; López, F.J.; Peñuela, G.A. Monitoring pharmaceuticals and personal care products in reservoir water used for drinking water supply. *Environ. Sci. Pollut. Res.* **2017**, *24*, 7335–7347, doi:10.1007/s11356-016-8253-1.
12. Goel, A.; Nene, S.N. Modifications in the Phenolphthalein Method for Spectrophotometric Estimation of Beta Cyclodextrin. *Starch-Stärke* **1995**, *47*, 399–400, doi:10.1002/star.19950471006.
13. Higuti, I.H.; Da Silva, P.A.; Papp, J.; Okiyama, V.M.D.E.; De Andrade, E.A.; Marcondes, A.D.A.; Nascimento, A.J.D. Colorimetric determination of alpha and beta-cyclodextrins and studies on optimization of CGTase production from B. firmus using factorial designs. *Braz. Arch. Biol. Technol.* **2004**, *47*, 837–841, doi:10.1590/s1516-89132004000600001.
14. de Souza, Ícaro F.; Petri, D.F. β-Cyclodextrin hydroxypropyl methylcellulose hydrogels for bisphenol A adsorption. *J. Mol. Liq.* **2018**, *266*, 640–648, doi:10.1016/j.molliq.2018.06.117.
